# Converging survival trends in non-small cell lung cancer patients with and without brain metastasis receiving state-of-the-art treatment

**DOI:** 10.1007/s11060-024-04562-0

**Published:** 2024-02-07

**Authors:** Itamar Averbuch, Roi Tschernichovsky, Shlomit Yust-Katz, Ofer Rotem, Dror Limon, Noga Kurman, Oded Icht, Daniel Reinhorn, Mor Moskovitz, Ekaterina Hanovich, Alexandra Benouaich-Amiel, Tali Siegal, Alona Zer, Omer Gal

**Affiliations:** 1 Davidoff Cancer Center, Rabin Medical Center– Beilinson Hospital, 39 Jabotinsky St, Petach Tikva, 4941492 Israel; 2 Faculty of Medicine, Tel Aviv University, Tel Aviv, 6997801 Israel; 3 Department of Molecular Cell Biology, Weizmann Institute of Science, Rehovot, Israel; 4 Neuro-Oncology Unit, Davidoff Cancer Center at Rabin Medical Center, Petach Tikva, 4941492 Israel; 5 Faculty of Medicine, Hebrew University, Jerusalem, Israel; 6 Fishman Oncology Institute, Rambam Health Care Campus, Haifa, Israel

**Keywords:** Non small cell lung cancer, Brain metastasis, Prognosis

## Abstract

**Introduction:**

Historically, patients with brain metastasis (BM) have been excluded from clinical trials investigating treatments for non-small cell lung cancer (NSCLC) due to their unfavorable prognosis. Advanced treatments have increased survival prospects for NSCLC patients with BM. This study evaluated the life expectancy of NSCLC patients with and without BM in the context of contemporary treatments.

**Methods:**

Outcome data were collected for patients with advanced NSCLC attending a tertiary medical center between 2015 and 2020. Patients were stratified according to BM status and compared for overall survival (OS) using log-rank and Cox regression analyses.

**Results:**

The cohort included 360 patients with NSCLC of whom 134 (37.2%) had BM. Most (95%) of cases of BM developed within the first two years: 63% at diagnosis, 18% during the first year, 14% during the second year. There was no significant difference in OS between patients without BM and those with BM (median 23.7 vs. 22.3 months, HR = 0.97, *p* = 0.82); patients with BM and a targetable or non-targetable mutation (40.2 vs. 31.4 months, HR = 0.93, *p* = 0.84, and 20.7 vs. 19.87 months, HR = 0.95, *p* = 0.75, respectively); and patients with symptomatic BM (23.7 vs. 19.8 months, HR = 0.95, *p* = 0.78). Treatment for BM (95% of patients) consisted of stereotactic radiosurgery or tyrosine kinase inhibitors, with corresponding intracranial control rates of 90% and 86%.

**Conclusion:**

The results imply that the presence of BM has no impact on the prognosis of NSCLC. The practice of excluding NSCLC patients with BM from clinical trials warrants reconsideration.

**Supplementary Information:**

The online version contains supplementary material available at 10.1007/s11060-024-04562-0.

## Introduction

Brain metastasis (BM) is a common complication of cancer. Approximately 24.2 new diagnoses occur annually per 100,000 persons, and at autopsy, 20–40% of all patients with metastatic cancer have BM [1, 2]. Left untreated, BM can have severe consequences, such as neurological deficits, seizures, cognitive impairment, decreased quality of life, coma, and death. Historically, BM have been associated with poor prognosis. An outcome analysis of patients treated for BM with different approaches found that 28% died within 30 days from treatment onset, and an additional 39% died within 60 days [3]. The incidence of BM will likely continue to rise due to the integration of brain imaging as part of the screening and staging process of cancer and the use of improved imaging techniques such as magnetic resonance imaging (MRI). Advancements in systemic treatments may also contribute to an increase in incidence, as patients with metastatic disease live longer and therefore have a higher chance of acquiring BM [4, 5]. 

Lung cancer is one of the most common solid malignancies that spread to the central nervous system (CNS). Two large series of lung cancer reported BM rates of 16–20% [6, 7]. Owing to their unfavorable prognosis, patients with non-small cell lung cancer (NSCLC) and BM have been historically excluded from most clinical trials to avoid potential underestimation of treatment benefits [8]. Trials that did not exclude patients with BM typically specified that the metastases should be asymptomatic, and patients should be off steroids. This suggests that BM may have been previously treated, creating a difficulty in precisely evaluating the intracranial effects of the exemend drug.

The ability of tumor cells to reach and grow in the CNS requires genetic or epigenetic alterations [6]. While comprehensive data on genetic alterations facilitating CNS penetration is limited, correlation data exists between the incidence of brain metastases (BM) and specific mutations in the anaplastic lymphoma kinase (*ALK*) and epidermal growth factor receptor (*EGFR*) genes. A study of 543 patients with advanced NSCLC found that 39.2% of those with *EGFR* mutations had BM compared to 28.2% of those without mutations [9]. In the ALEX trial comparing alectinib with crizotinib as the initial treatment for *ALK* fusion NSCLC, 40% of patients had BM at the time of inclusion into the study [10]. 

Recent advancements in targeted therapy, immunotherapy, and stereotactic radiosurgery (SRS) may improve survival for patients with BM. Studies using the Graded Prognostic Assessment (GPA) score provide a promising example of this potential. The GPA is designed to forecast the prognosis of patients with BM based on different parameters. For patients with NSCLC, the initial report published in 2012 showed a median survival of 7 months when BM was present. However, a 2020 update reported an extended median survival of 15 months. This update also added mutations in *EGFR* and *ALK* as significant prognostic factors that should be included in the GPA calculation [11, 12]. A recent analysis of causes of death in patients with lung cancer and BM found that only one-third died of BM, while the rest died of systemic disease progression [13]. 

The objective of this study was to assess the outcome of patients with NSCLC and BM in the current era of emerging CNS-active medications and focal radiotherapy and compare it to life expectancy of patients without CNS disease. The study aimed to investigate the hypothesis that, due to advancements in both systemic and local treatments, the prognosis of patients with BM is comparable to that of patients without BM, challenging conventional assumptions.

## Materials and methods

### Patients and design

The study population consisted of consecutive patients treated for metastatic NSCLC at the Rabin Medical Center, a tertiary university medical center, between January 2015 and August 2020. Data were extracted retrospectively from a database of all patients with comprehensive genomic profiling. exclusion criteria included lack of brain imaging or lack of demographic data. Patients were stratified based on the presence or absence of BM and their outcomes were subsequently compared.

### Data collection

The following data were collected: demographics, smoking history, tumor histology, commercial next-generation sequencing data, disease stage including brain metastasis, Eastern Cooperative Oncology Group (ECOG) performance status (PS) at the diagnosis of stage IV NSCLC, presenting symptoms, systemic and local treatment,, overall survival (OS), and presumed cause of death. Intracranial response rate (iRR) was defined as the percentage of patients who achieved either complete or partial intracranial response. The intracranial control rate (iCR) was defined as the percentage of patients who achieved any response or had stable disease on follow-up imaging studies. Programmed death-ligand 1 (PDL-1) Expression levels were reported as tumour proportion score (TPS) and classified as either negative (< 1%), low-positive (1–49%) or positive (≥ 50%). Overall survival (OS) was defined as the interval from diagnosis of stage IV disease to the time of death or last event.

### Statistical analysis

All statistical analyses were performed using SPSS© software, version 25 (IBM Corp., Armonk, NY). The correlation of BM status with categorical clinicopathologic variables was analyzed using chi-square test of independence, and with continuous clinical and pathological variables, using t-test for independent samples. Results are expressed as median. Kaplan-Meier and log-rank tests were run to determine differences in OS by BM status. A Cox regression model was formulated to quantify the hazard ratio (HR) for survival under various treatment modalities by BM status. A *p* value of < 0.05 was considered statistically significant.

## Results

**Fig. 1:**
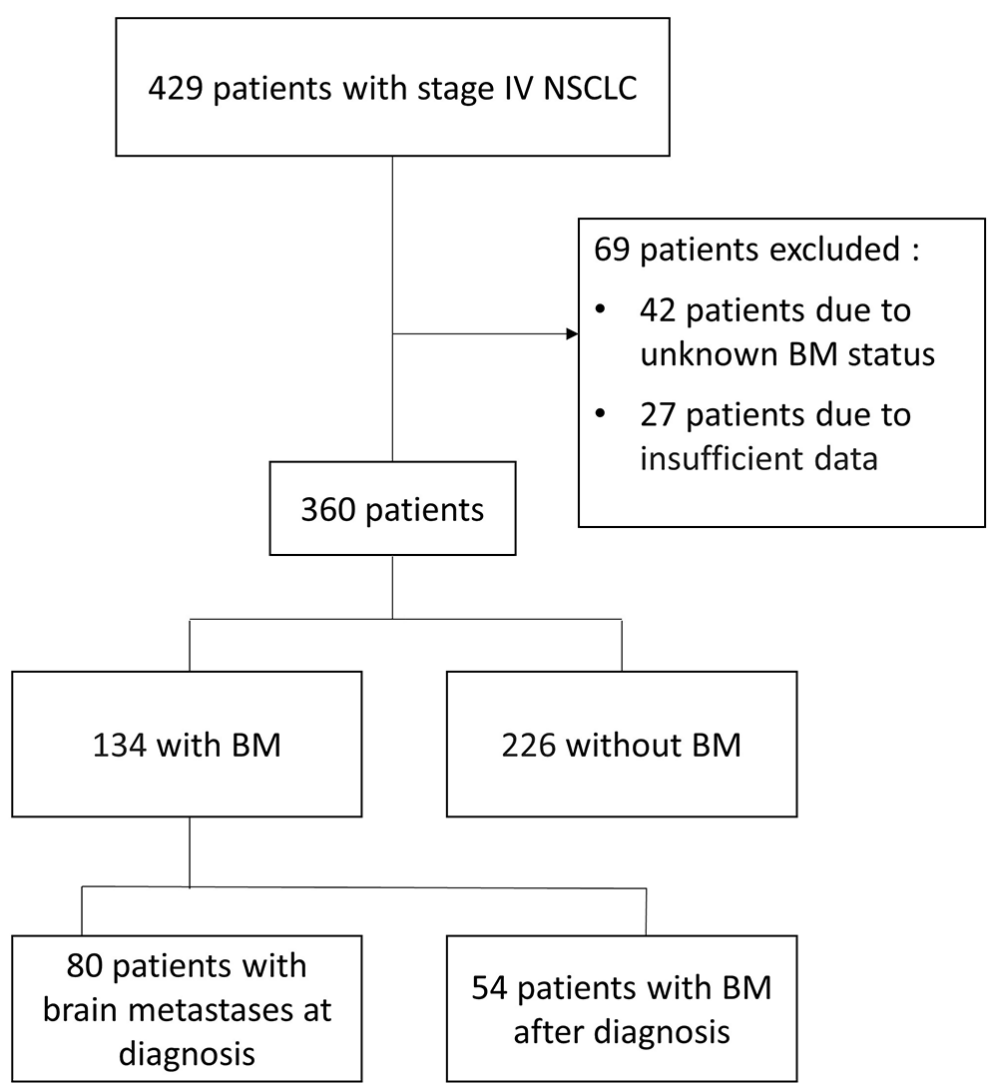
Chart flow

Of 429 patients with advanced NSCLC identified during the study period, 69 were excluded for lack of brain imaging data (*n* = 42) or insufficient clinical and epidemiological data (*n* = 27) (Fig. 1). The final cohort consisted of 360 patients. In a median follow-up of 50 months, 134 (37.2%) were diagnosed with BM and 226 (62.7%) without BM. The median age was 66 years at the diagnosis of stage IV disease. The group with BM contained a higher proportion of women (42.7% vs. 32.1%, *p* = 0.04) and was significantly younger (61.5 vs. 69 years, *p* < 0.001). BM were more often associated with adenocarcinoma than with squamous and adenosquamous histologies (*p* = 0.013). Data on PD-L1 expression were available for 139 patients. Among patients without BM, 45.7% exhibit a negative PDL-1 expression, 24.5% show low-positive and 29.8% demonstrate positive expression. For patients with BM, the proportions are 37.8%, 17.8%, and 44.4% for these respective categories. Other baseline characteristics are listed in Table 1. Data regarding the symptoms present at the onset of BM were available for 123 out of 134 patients diagnosed with BM. Among them, 58 (47.2%) were symptomatic, and 65 (52.8%) were asymptomatic. Baseline characteristics are listed in Table 1e.

**Table 1:** Background, tumor, and treatment characteristics of patients with NSCLC stratified by presence of brain metastasis (*n* = 360)

Characteristic	No BM (%)	BM (%)
**Sex (*n*** **= 354)**		
Male	129 (57.8)	61 (46.6)
Female	94 (42.2)	70 (53.4)
Age at stage IV (yr), mean (*n* = 325)	69	61.5
**Smoking (*n*** **= 293)**		
Never	69 (37.3)	43 (39.8)
Former/current	116 (62.7)	65 (60.2)
**ECOG (*n*** **= 192)**		
0–1	97 (80.8)	58 (80.6)
2–4	23 (19.2)	14 (19.4)
**Histology (*n*** **= 302)**		
Adenocarcinoma	163 (87.2)	112 (97.4)
Squamous carcinoma	20 (10.7)	2 (1.7)
Adenosquamous carcinoma	4 (2.1)	1 (0.9)
**PD-L1 expression (*n*** **= 139)**		
Negative (< 1%)	43 (45.7)	17 (37.8)
Low positive (1–49%)	23 (24.5)	8 (17.8)
Positive (50%)	28 (29.8)	20 (44.4)
**Targetable mutations (*n*** **= 360)**		
No	199 (88.1)	110 (82)
*EGFR* Exon 19 Deletion	16 (7.1)	10 (7.5)
*EGFR* L858R	8 (3.5)	8 (6)
*EML4-ALK* fusion	3 (1.3)	6 (4.5)
**First-line systemic treatment (*n*** **= 257)**		
Chemotherapy	84 (52.2)	50 (52.1)
Immune checkpoint inhibitors	32 (19.9)	19 (19.8)
Chemotherapy-immune checkpoint inhibitors	32 (19.9)	9 (9.4)
Tyrosine kinase inhibitors	13 (8.1)	18 (18.6)

BM, brain metastasis; ECOG, Eastern Cooperative Oncology Group

Values are expressed as n(%) unless otherwise indicated

**Fig. 2:**
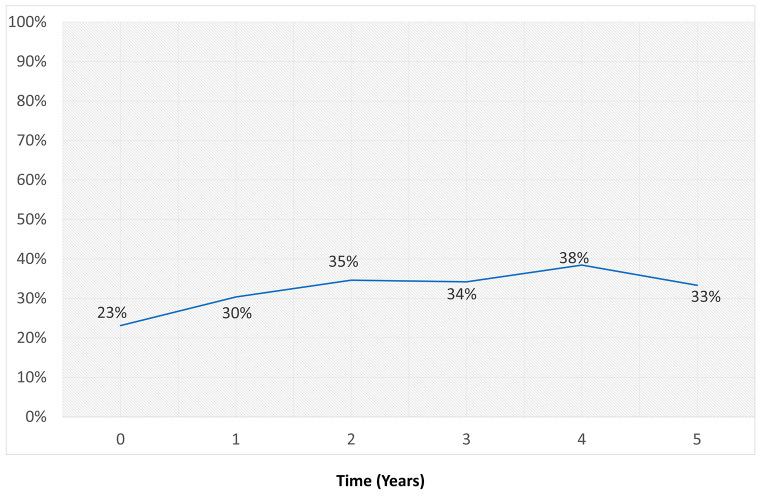
The ratio of patients with brain metastases among the overall surviving patients at yearly intervals following the diagnosis of NSCLC

**Fig. 3:**
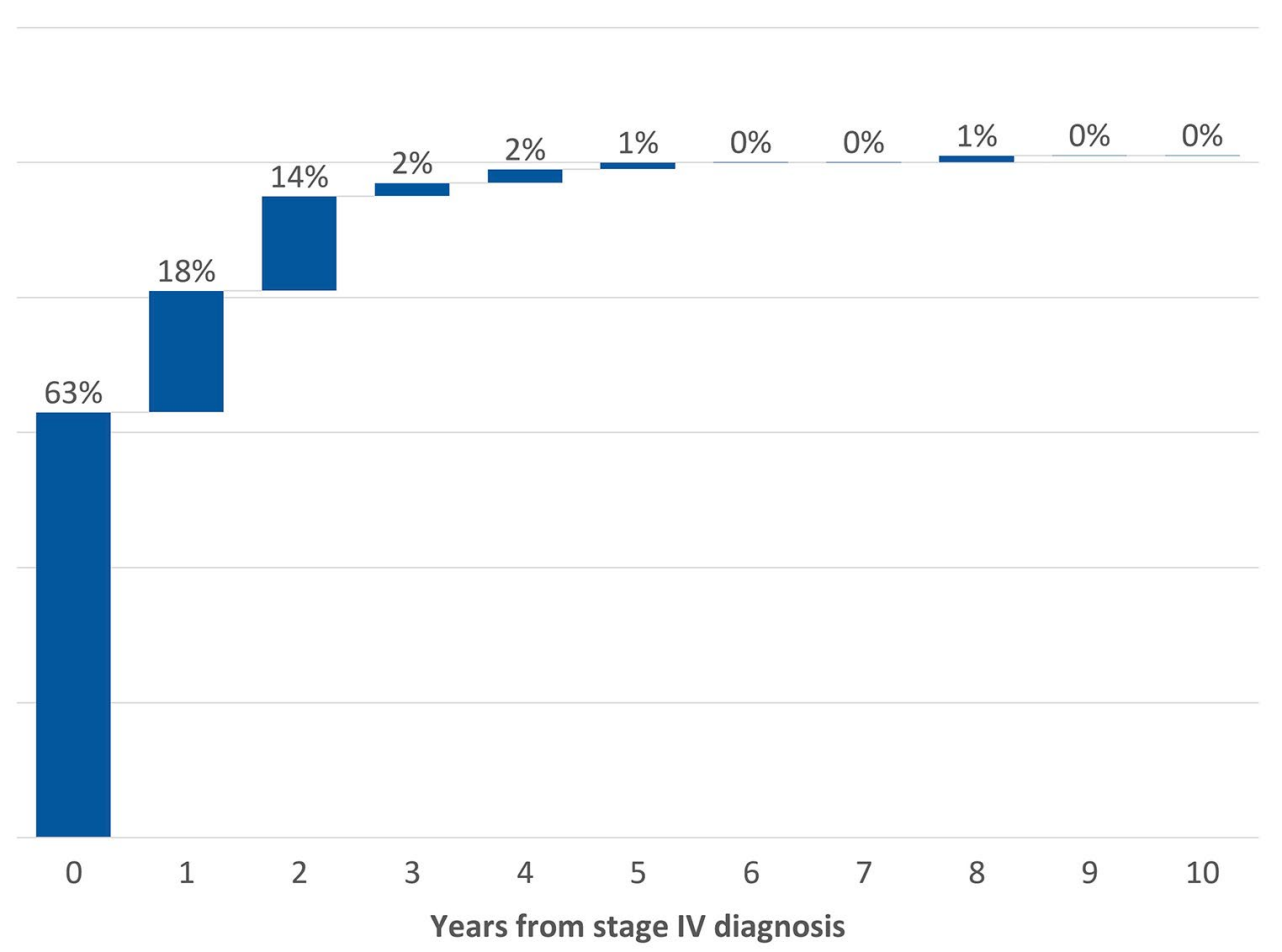
The yearly rate of patients developing brain metastases out of the entire group of patients with BM

Among the 134 patients with BM, 80 (59.7%) had BM at the time of stage IV disease diagnosis, representing 23% of the entire cohort (Fig. 1). In the remainder 54 patients, BM developed during the course of the disease. After one year of follow-up, 30.0% of the surviving patients had BM, 34.4% of the surviving patients at 2 years, 33.8% at 3 years, and 37.5% at 4 years (Fig. 2). In 95% of affected patients, BM developed within the first 2 years of diagnosis of stage IV NSCLC: 63% at diagnosis, 18% during the first post diagnosis year, and 14% during the second year (Fig. 3).

**Fig. 4:**
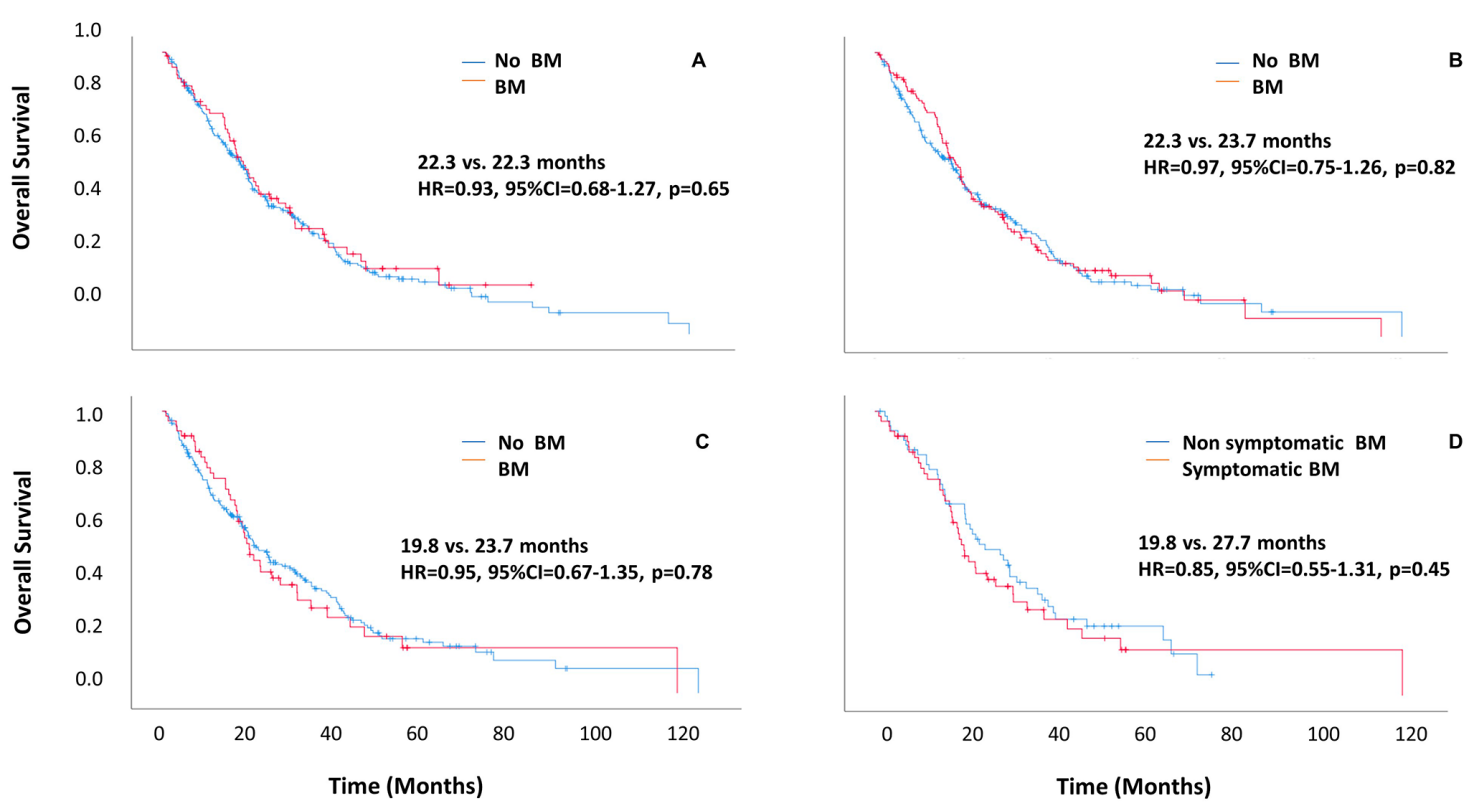
Overall survival by BM status. (**A**) Patients with BM at diagnosis of NSCLC compared to patients without BM. (**B**) Patients with BM at any time during the disease course compared to patients without BM. (**C**) Patients with symptomatic BM compared to patients without BM. (**D**) Patients with symptomatic BM compared to patients with non-symptomatic BM

OS did not differ significantly between BM patients and non-BM patients. This lack of difference was maintained when comparing patients with or without BM at diagnosis (median OS: 22.3 vs. 22.3 months, HR = 0.93, 95%CI = 0.68–1.27, *p* = 0.65) and patients with or without BM at any stage during the disease course (22.3 vs. 23.7 months, HR = 0.97, 95%CI = 0.75–1.26, *p* = 0.82). No significant difference in OS was found between patients without BM and patients with symptomatic BM (23.7 vs. 19.8 months, HR = 0.95, 95%CI = 0.67–1.35, *p* = 0.78) and between patients with symptomatic and non-symptomatic BM (19.8 vs. 27.7 months, HR = 0.85, 95%CI = 0.55–1.31, *p* = 0.45) (Fig. 4).

**Fig. 5:**
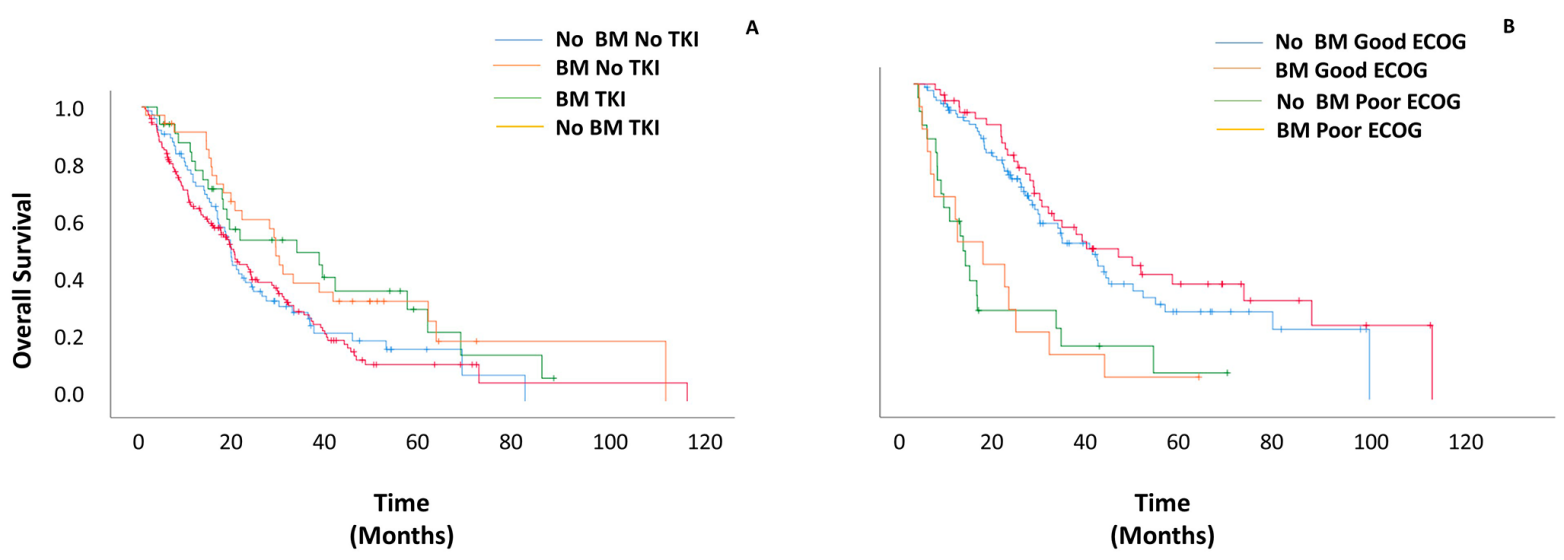
Overall survival stratified by brain metastasis status, performance status, and TKIs status. (**A**) Comparison of TKI-Treated or non-TKI-Treated patients with and without BM. (**B**) Comparison of patients with Good/Poor ECOG performance status with and without BM

51 patients had a targetable mutation identified in their tumors. 45 of them were treated with targeted therapy and 6 with non-specific systemic therapy. OS was significantly better in patients with a targetable mutation treated with tyrosine kinase inhibitors (TKIs) than in patients without a targetable mutation treated with non-specific systemic therapy (34.5 vs. 20 months, HR = 1.87, 95% CI = 1.3–2.8, *p* = 0.002). On subgroup analyses by treatment, the absence or presence of BM did not affect survival in patients with targetable mutations treated with TKIs (40.2 vs. 31.4 months, HR = 9.3, 95%CI = 0.44–1.94, *p* = 0.84); in patients without targetable mutations treated with non-specific systemic therapy (20.7 vs. 19.87 months, HR = 0.95, 95%CI = 0.69–1.3, *p* = 0.75). (Fig. 5); and in patients who received first-line treatment with chemotherapy (19.8 vs. 21.67 months, HR = 0.85, 95%CI = 0.58–1.2, *p* = 0.41) or immunotherapy and chemo-immunotherapy (23.1 months vs. 24.1 months, HR = 0.98, 95%CI = 0.74–1.3, *p* = 0.89). In a stratification of the immunotherapy and chemo-immunotherapy cohort according to PDL-1 status, the presence of BM did not affect survival in the PDL-1 negative (18.3 for no BM vs. 18.4 months for BM, HR = 1.2, 95%CI = 0.69–2.11, *p* = 0.51) or positive (37.7 for no BM vs. 31.4 months for BM, HR = 0.78, 95%CI = 0.35–1.87, *p* = 0.58) groups.

Adenocarcinoma histology was present in 275 patients, and squamous cell carcinoma in 22. Among those with adenocarcinoma, 163 had no brain BM, while 112 did. In the squamous cell carcinoma group, 20 had no BM, and 2 did. In subgroup analyses of patients with adenocarcinoma, the presence of BM did not affect survival. (median survival: 20.7 for no BM vs. 23.1 months for BM, HR = 0.89, 95%CI = 0.67–1.19, *p* = 0.42)

Patients with an ECOG score of 0 or 1 had a significantly better median OS than patients with a score ≥ 2 (27.7 vs. 8.1 months, HR = 3.2, 95%CI = 2.1–4.8, *p* < 0.001). On subgroup analysis, compared with patients without BM, the presence of BM had no effect on median OS in patients with a poor ECOG PS (7.2 vs.8.1 months, HR = 1.03, 95%CI = 0.51–2.1, *p* = 0.93) or in patients with a good ECOG PS (28.4 months vs. 24.4 months, HR = 0.8, 95%CI = 0.52–1.3, *p* = 0.33) (Fig. 5B).

Data on specific treatments for BM were available for 128/134 patients (96%). Ninety-five patients (74.2%) underwent brain surgery and/or radiation, including SRS (60.9%), whole-brain irradiation (25%), and neurosurgical intervention (10%); 21 patients (16.4) received local treatment with more than one modality. Among the remaining 33 patients, 12 (36.4%) received TKIs as first-line systemic treatment.

Data on response to CNS treatment were available for 52 patients who received SRS and 22 who received TKIs. In the SRS subgroup, iRR was 73% and iCR was 90% according to the first follow up brain MRI scan. Corresponding values in the TKI subgroup were iRR 50% and iCR 86%. Assessment of the 12 patients diagnosed with BM who received TKIs as first-line treatment yielded a iRR of 75% and iCR of 100%.

Cause of death was available in 75 patients with BM (56.0%): progressive CNS disease in 10%, progressive systemic disease or toxicity in 10%, and combined systemic and CNS disease in 36%.

## Discussion

The results of this study support the hypothesis that in the current era of advanced systemic and local treatment for NSCLC, the presence of BM does not impact prognosis. This hypothesis holds true across various subgroups, including patients with symptomatic BM, those with targetable mutations treated with TKIs, and individuals with either good or poor ECOG PS status. Additionally, it remains valid across different types of first-line treatments.

Prior to the introduction of targeted therapies and immune-check blockade (ICB) drugs [14], chemotherapy was the primary treatment for NSCLC. Patients with BM faced a dismal prognosis due to the lack of effectiveness of traditional treatment in the CNS [15]. In the KEYNOTE 042 trial, patients with advanced or metastatic NSCLC treated with pembrolizumab exhibited a median OS of 20 months [16]. In a Phase II trial that examined CNS response in NSCLC, among PD-L1 positive patients, the response rate for untreated BM was 29.7% [17]. In our ICB cohort, median OS was 24.1 months in patients without BM and 23.1 months in patients with BM, corresponding to survival rates observed in the KEYNOTE 042.

Regarding targeted treatment, the ALEX study showed an intracranial response rate of 81% and median duration of intracranial response of 17.3 months in patients treated with alectinib [10]. The FLAURA trial examined the effectiveness of osimertinib in treatment-naïve patients with *EGFR*-mutated NSCLC and measurable CNS lesions. The iRR was 91% in patients treated with osimertinib and 68% in patients in the control group [18]. Median OS in the osimertinib group was 38.6 months [19]. Although the iRR to TKIs in our study was only 50%, further analysis of patients with BM who received TKIs as first-line treatment, similar to the FLAURA trial, yielded a comparable iRR of 75% and iCR of 100%. Median OS in TKI-treated patients in the present cohort was 34.5 months.

In addition to improvements in systemic treatments, innovative technologies have also enhanced local treatment options. Thus, SRS may be used to treat nonresectable lesions that are either deep seated or located in eloquent brain areas [20]. A study examining predictors of response to SRS treatment reported local control rates of 91.2% on follow-up imaging, 88.6% at 1 year, and 77.2% at 2 years [21]. These findings are in line with the 73% iRR and 90% iCR observed in our cohort. An updated GPA study from 2020 found that in patients with NSCLC adenocarcinoma, median survival was 14 months in those treated with SRS alone and 31 months in those treated with surgery and SRS. The median survival ranged from 5 to 46 months [8]. As the prognostic assessment was conducted retrospectively, conclusions about causality and the specific benefits of SRS treatment could not be drawn. However, one possible explanation for the relatively long median survival of BM patients in our cohort, which was 22.3 months, could be attributable to the use of SRS in a considerable proportion of the cohort (60.9%), with another 10.2% undergoing neurosurgical intervention.

An additional factor that could potentially improve prognosis is the inclusion of MRI screening as part of the initial evaluation [22]. This may make it possible to treat brain metastases earlier using less toxic options [23]. 

In this report, we demonstrated that squamous cell carcinoma patients tend to exhibit a lower percentage of BM compared to adenocarcinoma patients. Given that adenocarcinoma patients generally have a better prognosis [24], it is plausible that the absence of a survival difference based on BM status in the overall cohort results from adenocarcinoma patients with BM having a better prognosis than squamous cell carcinoma SCC patients without BM. To address this hypothesis, we conducted a BM based survival analysis for adenocarcinoma patients, which showed no significant difference. Therefore, it does not seem that the lack of difference came from this hypotethis. Unfortunately, we couldn’t perform a similar analysis for squamous cell carcinoma due to a limited number of patients with BM (*n* = 2).

Another notable finding is the proportion of patients with BM during follow-up. The total number of patients in whom BM developed grew in the first 3 years of follow-up but remained stable thereafter. It is generally accepted that BM can arise at any stage of the disease, and the longer the patient lives, the greater the likelihood that BM will develop [2]. Our results are counter to this notion, suggesting that overall, the proportion of patients with BM remains relatively stable and that BM will not develop in most patients even over long-term surveillance.

Although most current phase III studies accept patients with treated, controlled, asymptomatic BM [23], a growing number continue to exclude patients with symptomatic, untreated, or uncontrolled BM [25]. Given the limited penetration of many drugs into the CNS, it is reasonable to expect that patients starting experimental treatments will also receive standard of care brain-directed treatment. However, with a HR of 0.95 and time difference of less than 4 months, our data contradict the claim that patients with neurological symptoms have a poorer prognosis. Therefore, we believe there may not be a compelling reason to exclude patients with symptomatic disease from participating in clinical trials. Their inclusion would also avoid an inherent bias in these trials, as in clinical practice, these patients are not disqualified from receiving treatment. Future studies are needed to investigate potential differences in prognosis between patients with symptomatic BM, asymptomatic BM, and no BM.

The present study has several strengths, including a comprehensive dataset and detailed information on clinical status, tumor molecular profiling, and treatment response for most of the patients. The study population was diverse, and most patients received state-of-the-art standard of care. The main limitations of the study are the retrospective design and single-center setting.

In conclusion, our data indicate that among NSCLC patients, the prognosis is comparable between those with and without brain metastases. This finding should encourage researchers to reevaluate the inclusion of NSCLC patients with brain metastases in clinical trials.

### Electronic supplementary material

Below is the link to the electronic supplementary material.

Supplementary Material 1

## Data Availability

The datasets generated during and/or analysed during the current study are available from the corresponding author on reasonable request.

## Abbreviations

*ALK*: anaplastic lymphoma kinase
BM: brain metastasis
CNS: central nervous system
ECOG: Eastern Cooperative Oncology Group
*EGFR*: epidermal growth factor receptor
GPA: Graded Prognostic Assessment
HR: hazard ratio
iCR: intracranial control rate
MRI: magnetic resonance imaging
NSCLC: non-small cell lung cancer
OS: overall survival
PS: performance status
iRR: intracranial response rate
SRS: stereotactic radiosurgery
TKIs: tyrosine kinase inhibators
