# Supplementary material for: Converging survival trends in non-small cell lung cancer patients with and without brain metastasis receiving state-of-the-art treatment
Source: J Neurooncol. 2024 Feb 7;166(3):461–9. doi: 10.1007/s11060-024-04562-0 (PMC10876498; doi:10.1007/s11060-024-04562-0)
Supplement: Supplementary file 1 — Supplementary Material 1 [file 11060_2024_4562_MOESM1_ESM.docx]

Supplementary material

|  |  | Non Sympthomatic BM (%) | Sympthomatic BM (%) | p-value |
| --- | --- | --- | --- | --- |
| **Sex** | Male | 28 (43) | 33 (57) | 0.126 |
|  | Female | 37 (57) | 25 (43) |  |
| **Age at Stage IV (mean)** |  | 62.4 | 61.7 | 0.21 |
| **Smoking** | Never | 32 (57) | 11 (22) | <0.001 |
|  | Former/Current | 24 (43) | 39 (78) |  |
| **ECOG** | 0-1 | 24 (69) | 31 (91) | 0.02 |
|  | 2-4 | 11 (31) | 3 (9) |  |
| **Histology** | Adenocarcinoma | 55 (97) | 50 (98) | 0.23 |
|  | Squamous | 2 (3) | 0 (0) |  |
|  | Adenosquamous | 0 (0) | 1 (2) |  |
| **PD-L1 expression** | Negative (<1%) | 10 (56) | 7 (26) | 0.11 |
|  | Low Positive (1-49%) | 3 (2) | 5 (18) |  |
|  | Positive (≥50%) | 5 (3) | 15 (56) |  |

Table1e Background and tumor charactericties of NSCLC patients with brain metastasis stratified to symptomatic and non-symptomatic BM (n=123)

BM, brain metastasis; ECOG, Eastern Cooperative Oncology Group

Values are expressed as n(%) unless otherwise indicated.
